# Observation of Mott instability at the valence transition of *f*-electron system

**DOI:** 10.1093/nsr/nwad035

**Published:** 2023-02-28

**Authors:** Haifeng Yang, Jingjing Gao, Yingying Cao, Yuanji Xu, Aiji Liang, Xiang Xu, Yujie Chen, Shuai Liu, Kui Huang, Lixuan Xu, Chengwei Wang, Shengtao Cui, Meixiao Wang, Lexian Yang, Xuan Luo, Yuping Sun, Yi-feng Yang, Zhongkai Liu, Yulin Chen

**Affiliations:** School of Physical Science and Technology, ShanghaiTech University, Shanghai 201210, China; Key Laboratory of Materials Physics, Institute of Solid State Physics, Chinese Academy of Sciences, Hefei 230031, China; Science Island Branch of Graduate School, University of Science and Technology of China, Hefei 230026, China; Beijing National Laboratory for Condensed Matter Physics and Institute of Physics, Chinese Academy of Sciences, Beijing 100190, China; University of Chinese Academy of Sciences, Beijing 100049, China; Beijing National Laboratory for Condensed Matter Physics and Institute of Physics, Chinese Academy of Sciences, Beijing 100190, China; School of Physical Science and Technology, ShanghaiTech University, Shanghai 201210, China; ShanghaiTech Laboratory for Topological Physics, Shanghai 201210, China; State Key Laboratory of Low Dimensional Quantum Physics and Department of Physics, Tsinghua University, Beijing 100084, China; State Key Laboratory of Low Dimensional Quantum Physics and Department of Physics, Tsinghua University, Beijing 100084, China; School of Physical Science and Technology, ShanghaiTech University, Shanghai 201210, China; School of Physical Science and Technology, ShanghaiTech University, Shanghai 201210, China; State Key Laboratory of Functional Materials for Informatics, Shanghai Institute of Microsystem and Information Technology (SIMIT), Chinese Academy of Sciences, Shanghai 200050, China; State Key Laboratory of Functional Materials for Informatics, Shanghai Institute of Microsystem and Information Technology (SIMIT), Chinese Academy of Sciences, Shanghai 200050, China; School of Physical Science and Technology, ShanghaiTech University, Shanghai 201210, China; School of Physical Science and Technology, ShanghaiTech University, Shanghai 201210, China; ShanghaiTech Laboratory for Topological Physics, Shanghai 201210, China; State Key Laboratory of Low Dimensional Quantum Physics and Department of Physics, Tsinghua University, Beijing 100084, China; Key Laboratory of Materials Physics, Institute of Solid State Physics, Chinese Academy of Sciences, Hefei 230031, China; Key Laboratory of Materials Physics, Institute of Solid State Physics, Chinese Academy of Sciences, Hefei 230031, China; High Magnetic Field Laboratory, Chinese Academy of Sciences, Hefei 230031, China; Collaborative Innovation Center of Microstructures, Nanjing University, Nanjing 210093, China; Beijing National Laboratory for Condensed Matter Physics and Institute of Physics, Chinese Academy of Sciences, Beijing 100190, China; University of Chinese Academy of Sciences, Beijing 100049, China; Songshan Lake Materials Laboratory, Dongguan 523808, China; School of Physical Science and Technology, ShanghaiTech University, Shanghai 201210, China; ShanghaiTech Laboratory for Topological Physics, Shanghai 201210, China; School of Physical Science and Technology, ShanghaiTech University, Shanghai 201210, China; ShanghaiTech Laboratory for Topological Physics, Shanghai 201210, China; Department of Physics, University of Oxford, Oxford, OX1 3PU, UK

**Keywords:** valence transition, orbital-selective Mott transition, Kondo coupling, heavy fermions, strong correlations, ARPES, DMFT

## Abstract

Mott physics plays a critical role in materials with strong electronic correlations. Mott insulator-to-metal transition can be driven by chemical doping, external pressure, temperature and gate voltage, which is often seen in transition metal oxides with *3d* electrons near the Fermi energy (e.g. cuprate superconductor). In *4f*-electron systems, however, the insulator-to-metal transition is mostly driven by Kondo hybridization and the Mott physics has rarely been explored in experiments. Here, by combining the angle-resolved photoemission spectroscopy and strongly correlated band structure calculations, we show that an unusual Mott instability exists in YbInCu_4_ accompanying its mysterious first-order valence transition. This contrasts with the prevalent Kondo picture and demonstrates that YbInCu_4_ is a unique platform to explore the Mott physics in Kondo lattice systems. Our work provides important insight for the understanding and manipulation of correlated quantum phenomena in the *f*-electron system.

## Teaser

Towards the origin of the mysterious valence transition in Kondo lattices, orbital-selective Mott physics stands out unexpectedly.

## INTRODUCTION

**Figure 1.**
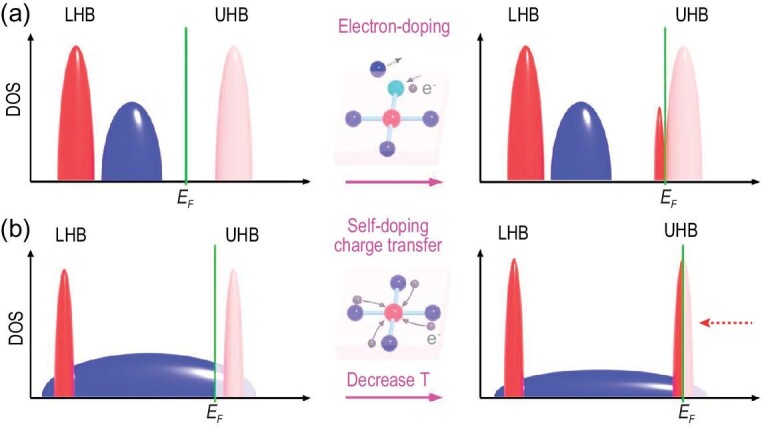
(a) Simplified sketch of doping electrons to an archetypical *3d* Mott insulator like cuprate superconductor (note the limitations of the simplifications). Doping electrons will create density of states at the upper Hubbard band (UHB), leading to a metallic state and the emergence of high-temperature superconductivity in cuprates. LHB, DOS and *E_F_* are short for lower Hubbard band, density of states, and Fermi energy, respectively. (b) Sketch of exotic temperature (T)-relevant self-doped orbital-selective Mott transition in *4f* Kondo lattice systems with strong Mott instability (the UHB is close to the *E_F_* before transition). The blue band represents a general conduction band. Due to the self-doping effect and charge transfer when decreasing T, the UHB moves to *E_F_* (as marked by the red arrow), realizing an orbital-selective Mott transition.

In materials with strong electron-electron correlations, Mott insulator plays a central role as the parent state of many intriguing properties such as unconventional superconductivity and quantum spin liquids [1,2]. An insulator-to-metal transition can usually be realized by controlling the band filling (e.g. with doping) or bandwidth (e.g. with external pressure) [1], driven by changing temperature or even applying gate voltage [1–3]. Such Mott physics is often seen in 3*d* transition metal oxides with strong correlations. Taking the cuprate family as an example, doping a Mott insulator with electrons will create a density of states at the upper Hubbard band (UHB) (Fig. 1a), leading to a metallic state and the emergence of high-temperature superconductivity [2]. In multi-orbital systems, orbital selective Mott transition has been proposed and observed in ruthenates (*4d*) [4] and iron-based superconductors (*3d*) [5,6]. In *5d* iridates, spin-orbit interaction plays a critical role in the formation of the *J*_eff_ = 1/2 Mott insulating ground state [7].

In comparison, Mott physics manifests itself in a more sophisticated way in *4f*-electron systems, because of the narrow bandwidth and strong competition of localization and itinerancy of *4f* electrons as well as complex many-body physics [8–11], which is different from common Kondo physics (e.g. Kondo hybridization induced band-gaps) and has rarely been explored experimentally. If one *f* band resides close to the Fermi level (*E_F_*), strong Mott instability exists in the sense that small perturbations can drive the system to Mott transition. Indeed, temperature-related localization-itinerancy competition may induce charge transfer and valence fluctuation that drag the *f* band to *E_F_*, thus forming a temperature-related self-doped orbital-selective Mott transition (Fig. 1b). In this paper, we verify such Mott instability and Mott transition does indeed exist and accompany the first-order iso-structural valence transition (VT) in YbInCu_4_, one of the most fascinating puzzles in strongly-correlated Kondo lattice systems [12–16].

YbInCu_4_ is the only stoichiometric compound exhibiting VT at ambient pressure with the transition temperature *T_V_* = 42 K [15,16], in contrast to the γ-α transition of cerium metal under pressure [12]. Since being first observed in the 1980s [14], plenty of experimental efforts have been invested and consistently revealed sudden changes of various physical properties at VT [15–30], in stark contrast to the slow crossover behaviors in common mixed-valence metals [10,11]. When cooling through *T_V_*, Yb valence suddenly drops from +2.9 to +2.74 [21] with magnetism switching from local-moment susceptibility of ∼4.5 ${u}_B$ to temperature-independent paramagnetism [16]. Meanwhile, the specific heat curve features a sharp peak at *T_V_*, indicating a first-order-type transition [17]. Correspondingly, the electrical resistivity and Hall coefficient decrease by one order of magnitude [15,18], implying dramatic changes in the electronic structures. Furthermore, it is generally believed that the transition is accompanied with a sudden change of the Kondo temperature (*T_K_*) from ∼20 K to ∼400 K [18,27], although the crystal symmetry is unchanged and the lattice volume increases only slightly by ∼0.5% [31,32].

Such dramatic contrast [15,17] poses a severe challenge to the theory. The conventional Kondo scenario and Mott scenario have been proposed [33], and various conjectures ascribed VT to either the combined effect of a quasi-gap in the density of states (DOS) [19,26,28,34,35] and a shift of *E_F_* (induced by charge transfer [23] or strong electron correlation [36]), lattice distortion [37], hybridization [22,28]/Coulomb interaction [33] between *f* and conduction electrons, or geometrical frustration [20]. Direct measurement of band structures near *E_F_* and their evolution across *T_V_* will greatly help uncover the origin of VT. However, such measurement has not be reported.

Here, we fill in this gap by conducting high-resolution synchrotron- and laser-based angle-resolved photoemission spectroscopy (ARPES) studies on electronic structures of YbInCu_4_. We observed the overall *E_F_*-adjacent band structures including both dispersive and two flat bands, and their evolution across VT for the first time. Our results are consistent with the expected valence change, but show no evidence of enhanced band hybridization below *T_V_* as expected for *T_K_* = 400 K within the conventional Kondo scenario. Instead, these results are qualitatively explained by our density functional theory (DFT) and dynamical mean-field theory (DMFT) calculations, and suggest a novel temperature-driven self-doped orbital-selective Mott transition across VT (Fig. 1b), thus rendering YbInCu_4_ an intriguing platform for the investigation of Mott physics in the *f*-electron system.

## RESULTS

**Figure 2.**
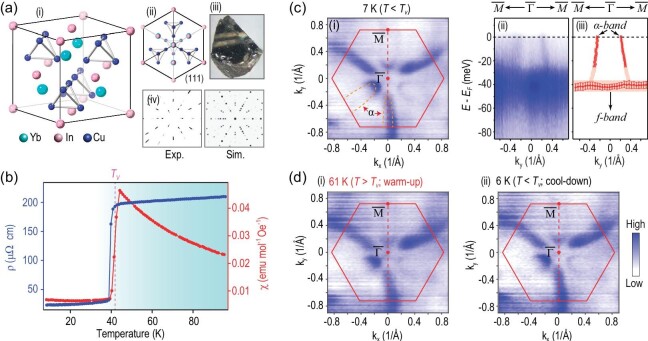
Basic characterization and overall electronic structure of YbInCu_4_. (a) Sketch of crystal structure of YbInCu_4_ (i) and its view along 〈111〉 direction (ii), optical image of typical single crystal (iii), measured (Exp.) and simulated (Sim.) Laue diffraction patterns of (111) surface (iv). (b) Temperature dependence of electrical resistivity (left) and magnetic susceptibility (right), both consistently showing abrupt changes at 42 K (valence transition temperature). (c) General band structure of YbInCu_4_ measured with horizontally polarized photons of 45 eV: Fermi surface map at 7 K (< *T_V_*) (i), high-symmetry band dispersions along the $\bar{M}$—$\bar{\Gamma }$—$\bar{M}$ direction (ii) and their fitting results by tracking peak maxima of energy/momentum distribution curves (EDC/MDCs) (iii). In (i), red hexagon represents the projected BZ on (111) surface and overlaid orange curves schematically represent ellipse pockets of the Fermi surface. (d) Fermi surface maps measured at 61 K (> *T_V_*) (i) and 6 K (< *T_V_*, cooled down again from 61 K) (ii).

YbInCu_4_ crystallizes in the face-centered-cubic (FCC) AuBe_5_ structure, in which In atoms form an FCC cage orderly filled with Yb atoms and Cu tetrahedrons (Fig. 2a). High-quality single crystals were synthesized by the InCu-flux method [31] (Fig. 2a(iii)) and show a sharp Laue diffraction pattern with three-fold rotation symmetry from the natural (111) surface (Fig. 2a(iv)). As the temperature decreases across VT, the electrical resistivity suddenly jumps with the concomitant switch from Curie–Weiss-type magnetic susceptibility to temperature-independent paramagnetism (Fig. 2b). The extracted transition temperature *T_V_* is ∼42 K (with transition width less than 2 K), in accordance with previous reports [16,18]. YbInCu_4_ crystals were successfully cleaved *in situ* to expose (111) surface for synchrotron- and laser-based (*hv* = 6.994 eV) ARPES measurements (Supplementary Material).

The measured overall band structure of YbInCu_4_ is plotted in Fig. 2c. Elliptical pockets encircling the $\bar{M}$ point (the projected Brillouin zone (BZ) is adopted for simplicity hereafter) are observed in the Fermi surface (Fig. 2c(i)). From the high-symmetry $\bar{M}$—$\bar{\Gamma }$—$\bar{M}$ direction cut, one can identify the dispersive bands (labelled as α bands) and flat band (labelled as *f* band) located ∼40 meV below *E_F_* (see Fig. 2c(ii) and 2c(iii) for the fitted dispersions). The α bands cross *E_F_* and form electron pockets of the Fermi surface. We further investigate the evolution of the Fermi surface constituted by α bands across the VT. When heating and cooling the sample through *T_V_*, we did not observe any evident expanding/shrinking or reconstruction of the α bands within our instrument resolutions, as revealed by the direct comparison of Figs. 2c(i) (7 K), 2d(i) (heating up to 61 K) and 2d(ii) (cooling down back to 6 K) (Supplementary Fig. S2). This is quite unexpected since the Fermi surface is supposed to change following the localization-itinerancy transition across *T_V_* according to the conventional Kondo picture [10].

**Figure 3.**
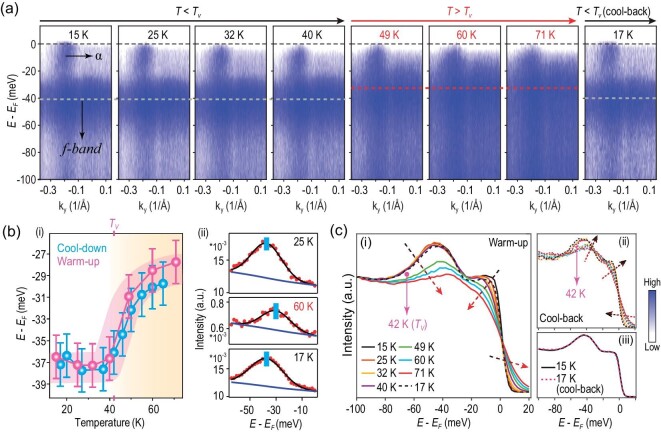
Detailed temperature-dependence of near-*E_F_* band structure measured with 6.994-eV laser-ARPES. (a) Temperature evolution of the $\bar{\Gamma } - \bar{M}$ dispersion (15 K$\to $71 K$\to $17 K). Overlaid dotted lines roughly mark binding energy positions of the *f* band. (b) Temperature-dependence of extracted binding-energy of the *f* band (i) and three representative fitting results (25 K, 60 K and 17 K) (ii) show shift-up of *f* band across *T_V_*. The EDC is extracted around *k_y_* = 0 and fitted with a Gaussian function. (c) Temperature-dependence of integrated EDCs including both α and *f* bands. Warm-up results (i) are well reproduced by a follow-up cool-back (ii). In (i) and (ii), EDCs of both warm-up and cool-back can be easily classified into two groups, well separated by *T_V_*, indicative of the valence transition. In (iii), integrated EDC at 15 K perfectly matches that of 17 K (cool-back) (area-normalization is used), again verifying our temperature-dependence results are reliable.

Further, we carefully investigated the dispersions of α and *f* bands to address the evolution of the VT-related electronic structure. Laser-based ARPES provides suitable photoemission cross sections so that both α/*f* bands could be clearly observed (and with high energy/momentum resolutions). We performed detailed temperature-dependent measurement of the $\bar{M}$—$\bar{\Gamma }$—$\bar{M}$ dispersion from 15 K to 71 K and then back to 17 K (Fig. 3, Supplementary Figs S3 and S4). One can apparently see that while the α band does not show evident differences across *T_V_*, the *f* band suddenly jumps towards *E_F_* when warming up across *T_V_* (Fig. 3a). We track the *f* band by fitting the energy distribution curve (EDC) around *k_y_* = 0 and could clearly observe an ∼9 meV energy jump towards *E_F_* (and far away from *E_F_* when cooling down again) (Fig. 3b). The reliability of our data is confirmed by systematic temperature-cycle measurements, as shown in Fig. 3c.

**Figure 4.**
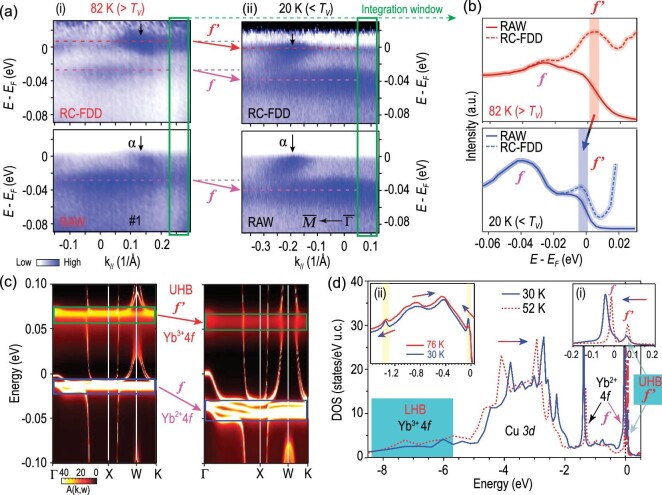
Mott instability and Mott transition across the VT. (a) (i) The *f*′ band exists ∼6 meV above *E_F_* at 82 K (> *T_V_*), as revealed by dividing the ARPES data along #1 (Supplementary Fig. S6) (lower panel) with energy-resolution-convolved Fermi–Dirac distribution function (RC-FDD) (upper panel). (ii) The *f*′ band moves to *E_F_* at 20 K (< *T_V_*) as revealed by dividing the ARPES data along $\bar{\Gamma }$—$\bar{M}$ (lower panel) with RC-FDD (upper panel). (b) Integrated EDCs (not including dispersive α bands; integration window marked by green rectangles in a) at 82 K (in red) and 20 K (in blue), clearly showing the *f*′ band shifts downward to *E_F_* when cooling through *T_V_*. (c) DFT + DMFT calculations of band dispersions at 52 K (> *T_V_*) (left) and 30 K (< *T_V_*) (right) with Yb *4f* bands marked. The *f*′ and *f* bands are verified to be *4f* bands of Yb^3+^ and Yb^2+^, respectively, both of which cannot be ascribed to Kondo resonance peaks. Note that the *f*′ band exhibits evident broadening reflecting strong self-energy effects, in contrast to the *f* band. Apparently, *f*′ and *f* bands move towards high-binding-energy regions when cooling through *T_V_*, agreeing with experimental results. (d) Calculations of density of states at 52 K (in red) and 30 K (in blue). Inset: (i) near-*E_F_* zoom-in of calculated density of states; (ii) integrated EDCs measured at 30 K and 76 K.

In addition to the *f* band, another flat band labelled as *f*′ is observed to reside at ∼6 meV above *E_F_* at 82 K (above *T_V_*), as is clearly revealed by dividing the spectrum with energy-resolution-convolved Fermi–Dirac distribution function (RC-FDD) (Fig. 4a(i)). At 20 K (below *T_V_*), the *f*′ band could also be found near *E_F_*(Fig. 4a(ii)). Such comparison suggests that accompanying the sudden jump-down of the *f* band, the *f*′ band moves downward as well to approach *E_F_* when cooling through *T_V_* (Figs. 4a and b, and Supplementary Fig. S5).

What could account for such VT-related band evolution? Such behavior could not be explained within the conventional Kondo picture. The VT is associated with abrupt increase of Kondo temperature *T_K_* from ∼20 K to ∼400 K [18,27]. For such a large *T_K_* value below *T_V_*, one would typically expect an indirect hybridization gap Δ*g* ∼ *T_K_* [10] ∼35 meV between dispersive α and *f/f*′ bands, which is quite large compared to our ARPES energy resolution (better than 5 meV using laser). Such change in the hybridization would lead to the bending of the conduction bands (α-bands in our case), which has been reported in typical heavy fermion systems such as CeCoIn_5_ whose *T_K_* = 6.6 K is even one order of magnitude smaller [38]. However, we do not observe any such signature across *T_V_* in our measurements (Figs. 2c, 3a and 4a). Our experiment contrasts sharply with the expectation for an abrupt change of *T_K_*, thus suggesting that the VT cannot be solely described by the conventional Kondo picture and the *f/f*′ band cannot be simply assigned as the Kondo resonance peak.

To gain more insights into the VT, we simulated band structures across *T_V_* using the full-potential linearized augmented plane-wave method in WIEN2k [39] combined with the DMFT implementation [40] (more details can be found in Supplementary Material). The *4f* occupation number (*N_f_*) is set to 13.3 for 30 K (below *T_V_*) and 13.1 for 52 K (above *T_V_*), respectively.

Our calculations qualitatively capture the key experimental results: it reproduces the *f* and *f*′ bands and their shift-down behaviors when cooling through *T_V_* (Fig. 4c) (note exact positions of *f* and *f*′ bands in calculations are slightly higher in energy due to potential numerical uncertainty). Analyses of the spectral weight show that Yb ions could be viewed as a mixture of Yb^2+^ and Yb^3+^ states (the true valence of Yb ions is close but not identical to +2 or +3 according to the simple ionic picture, and there is no spatial separation of Yb^2+^ and Yb^3+^ sites). A direct comparison with ARPES data suggests that the *f*-band (and its spin-orbital counterpart located at ∼ –1.3 eV) originates from Yb^2+^ (Fig. 4d and Supplementary Fig. S7), which can be well described by DFT with proper shift and renormalization. By contrast, the *f*′-band stems from the *4f* hole band (or UHB) of Yb^3+^ and exhibits evident broadening reflecting strong self-energy effects (Figs. 4c and d). Both bands move downwards with increasing valence but persist with increasing Coulomb interaction (see Supplementary Fig. S8), which cannot be of the Kondo resonance origin (as they persist at large Coulomb interaction that suppressed the Kondo scale) in line with discussions above (based on our detailed orbital analysis in Supplementary Fig. S10, *f* and *f*′ are clearly of Yb *4f* orbitals with *J* = 7/2). The LHB always resides at around –*U* and shifts according to the value of *U* (Supplementary Fig. S8), while the UHB *f*′-band is close to *E_F_* (Figs. 4a and b) so that the system exhibits strong Mott instability under small perturbations in the *4f* valence.

Indeed, the VT-induced charge transfer is seen to serve as a self-doping route to move down the *f*′-band to *E_F_* and realize the Mott transition. Upon cooling through *T_V_*, the sudden decrease of Yb valence (i.e. electrons are transferred to Yb from In/Cu) is directly reflected by the facts that peak shifts and spectral weight transfer of Yb and Cu occur in opposite directions, as revealed by ARPES integrated EDCs and calculated DOS (Fig. 4d) near *E_F_* below/above *T_V_*. Such drastic change of the spectra naturally reflects the valence change across *T_V_*, which would in turn trigger the Mott transition in the Yb^3+^ band. We note that electronic structure change induced by the self-doping is not a simple rigid-band shift, and the filling change (valence change) is directly manifested as the ratio change of the Yb^2+^ and Yb^3+^ spectral weights, or the change of the peak intensities of *f* and *f*′, and their shift-down behaviors when cooling the system through *T_V_*.

Taking all results together, we propose a temperature-relevant valence-change-driven orbital-selective Mott transition picture across the VT in YbInCu_4_, combining Fig. 1b and Fig. 4. Upon cooling through *T_V_*, the VT occurs with background electrons (mainly Cu *d* electrons) being transferred to Yb. Such valence-change-related self-doping changes the chemical environment of Yb ions and drags the Yb^3+^ UHB *f*′ (which is very close to *E_F_*) down to *E_F_*, thus contributing to orbital-selective Mott transition [41] of the Yb *4f* band near *E_F_* as well as abrupt changes of various physical properties in YbInCu_4_ (dispersive α band plays a less important role). Different from conventional Mott transition that is commonly bandwidth- or filling-controlled via external doping or pressure [1,42], the Mott transition in YbInCu_4_ is induced by self-doping. Furthermore, the system above *T_V_* is on the verge of an orbital-selective Mott transition and displays strong Mott instability, this can be easily perturbed to realize such transition. One possible driving force could be the strong Coulomb interaction between the *f* and other electrons [43], which also couples to the lattice and causes sudden structural change (as lattice volume increases by ∼0.5% across the VT). We did not mean to claim that the Kondo physics is completely absent; rather, the true *T_K_* is too small and the Kondo hybridization is too weak to have an evident effect on the band structure, and the physics of YbInCu_4_ is dominated by the Mott physics. We note that the Mott transition in YbInCu_4_ is similar to that of the iron-based superconductors, as they both occur when warming them up and the system still remains metallic [5,6]. However, detailed band structure changes are distinct: by increasing temperature, the iron-based superconductors are characterized by dramatic spectral weight reduction of the Fe *3d_xy_* orbital (while other orbitals remain itinerant) [5,6], whereas YbInCu_4_ is featured by a self-doping induced shift of the *f*′ band with Yb^3+^ origin. It is interesting to compare YbInCu_4_ with Ce-metal whose valence transition is triggered by pressure. In Ce, it is suggested that both Kondo physics and Mott physics coexist and act cooperatively [44], whereas in YbInCu_4_ the Mott physics dominates over the Kondo coupling.

## CONCLUSION

To summarize, we investigated the sharp VT of YbInCu_4_ with the combined efforts of ARPES measurements and DFT/DMFT calculations. We acquired general *E_F_*-adjacent band structure including dispersive α band and flat *f/f*′ bands, and their behaviors across VT, which cannot be explained solely within the conventional Kondo picture. Instead, we propose a self-doped Mott transition picture, as UHB *f*′ shifts downward to *E_F_* upon cooling through *T_V_* because of VT-associated charge transfer. We demonstrated that YbInCu_4_ provides a unique case to explore the Mott instability and subsequent Mott transition in strongly correlated Kondo *4f* systems. Although the large self-energy of *f*′ band in calculations cannot be directly confirmed by our ARPES, and calculated *f*′ band is slightly above *E_F_*, we stress the discovery of such an abrupt band evolution in a typical *f*-electron system is quite unexpected, and the driving force behind such evolution deserves further scrutiny by the community, while our results could provide a good starting point.

## METHODS

### Crystal growth

Single-crystals of YbInCu_4_ were grown in an InCu flux [31]. A 1 : 1 ratio of YbInCu_4_ to InCu was mixed as the starting material, and then placed in an Al_2_O_3_ crucible that was sealed under vacuum in a quartz tube. The material was heated up to 1400 K and kept for 5 hours, cooled down at a rate of 20 K/h to 1000 K, then cooled down to room temperature. Prior to ARPES studies, crystals were characterized by XRD (Supplementary Fig. S1a) and transport measurements (Fig. 2b of the main text).

### Laser- and synchrotron-based ARPES measurements

Fresh YbInCu_4_ surfaces were obtained by *in situ* cleaving of the crystals at low temperatures. As cleaved surfaces were commonly fractured and small (Supplementary Fig. S1c), ARPES systems with small beam spot are necessary to yield reliable band structure data. Laser-ARPES (6.994 eV) measurements were performed on home-built ARPES systems with energy resolution better than 5 meV. The system utilizes a Scienta DA-30 Analyzer and the base vacuum is 2.5E-11 mbar. Temperature-cycle experiments were carefully conducted and repeated. ARPES measurements were also carried out on synchrotron-based ARPES systems, e.g. BL I05 of the Diamond Light Source, BL 5–2 of Stanford Synchrotron Radiation Lightsource, BL10 of Advanced Light Source, SpectroMicroscopy of Elettra, BL03U of Shanghai Synchrotron Radiation Facility, and BL13U of National Synchrotron Radiation Laboratory Data, most of which have an overall energy resolution of 15 meV and angle resolution of 0.2°.

### DFT + DMFT calculations

Strongly correlated electronic band structure calculations were carried out using the full-potential linearized augmented plane-wave method in WIEN2k [39] combined with the DMFT implementation [40]. We have used the generalized-gradient approximation with the Perdew–Burke–Ernzerhof (GGA-PBE) exchange-correlation potential [45] and the spin-orbit coupling was included. A lattice constant of *a* = 7.150 Å and the Cu atomic position of (3/8, 3/8, 3/8) were adopted from the literature [46]. The muffin-tin radii (*R*_MT_) were set to 2.3 a.u. for Cu and 2.5 a.u. for Yb and In. The maximum modulus for the reciprocal vector *K*_max_ was chosen such that *R*_MT_*K*_max_ = 8.0. A Coulomb interaction *U* of 6.0 eV and a Hund coupling *J* of 0.7 eV were applied to all Yb *4f* orbitals according to experiment [47] and constrained LSDA estimate [48]. The double counting was subtracted with the nominal scheme. For the DMFT calculations, we have used the hybridization expansion continuous-time quantum Monte Carlo as the impurity solver [49]. To obtain high accuracy at low temperatures, 10^8^ CT-HYB steps were performed per processor on over 24 processors. The spectra were obtained via analytic continuation using the maximum entropy method [50].

## Supplementary Material

nwad035_Supplemental_File
