# Supplementary material for: Observation of Mott instability at the valence transition of f-electron system
Source: Natl Sci Rev. 2023 Feb 28;10(6):nwad035. doi: 10.1093/nsr/nwad035 (PMC10359067; doi:10.1093/nsr/nwad035)
Supplement: nwad035_Supplemental_File [file nwad035_supplemental_file.pdf]

SUPPLEMENTAL MATERIAL

Observation of Mott instability at the valence transition of *f*-electron system

H. F. Yang[1*], J. J. Gao[2,8*], Y. Y. Cao[3*],Y. J. Xu[3], A. J. Liang[1,4], X. Xu[5], Y. J. Chen[5], S. Liu[1], K. Huang[1], L. X. Xu[6], C. W. Wang[6], S. T. Cui[1], M. X. Wang[1,4], L. X. Yang[5], X. Luo[2], Y. P. Sun[2,9,10], Y.-F. Yang[3†], Z. K. Liu[1,4†], and Y. L. Chen[1,4,7†]

[1]*School of Physical Science and Technology, ShanghaiTech University, Shanghai 201210, China*
[2]*Key Laboratory of Materials Physics, Institute of Solid State Physics, Chinese Academy of Sciences, Hefei 230031, China*
[3]*Beijing National Laboratory for Condensed Matter Physics and Institute of Physics, Chinese Academy of Sciences, Beijing 100190, China*
[4]*ShanghaiTech Laboratory for Topological Physics, 201210 Shanghai, China*
[5]*State Key Laboratory of Low Dimensional Quantum Physics and Department of Physics, Tsinghua University, Beijing 100084, China*
[6]*State Key Laboratory of Functional Materials for Informatics, Shanghai Institute of Microsystem and Information Technology (SIMIT), Chinese Academy of Sciences, Shanghai 200050, China*
[7]*Department of Physics, University of Oxford, Oxford, OX1 3PU, UK*
[8]*Science Island Branch of Graduate School, University of Science and Technology of China, Hefei 230026, China*
[9]*High Magnetic Field Laboratory, Chinese Academy of Sciences, Hefei, 230031, China*
[10]*Collaborative Innovation Center of Microstructures, Nanjing University, Nanjing 210093, China*

[*] *These authors contributed equally to this work*
[†] *Email: yifeng@iphy.ac.cn, liuzhk@shanghaitech.edu.cn, yulin.chen@physics.ox.ac.uk*

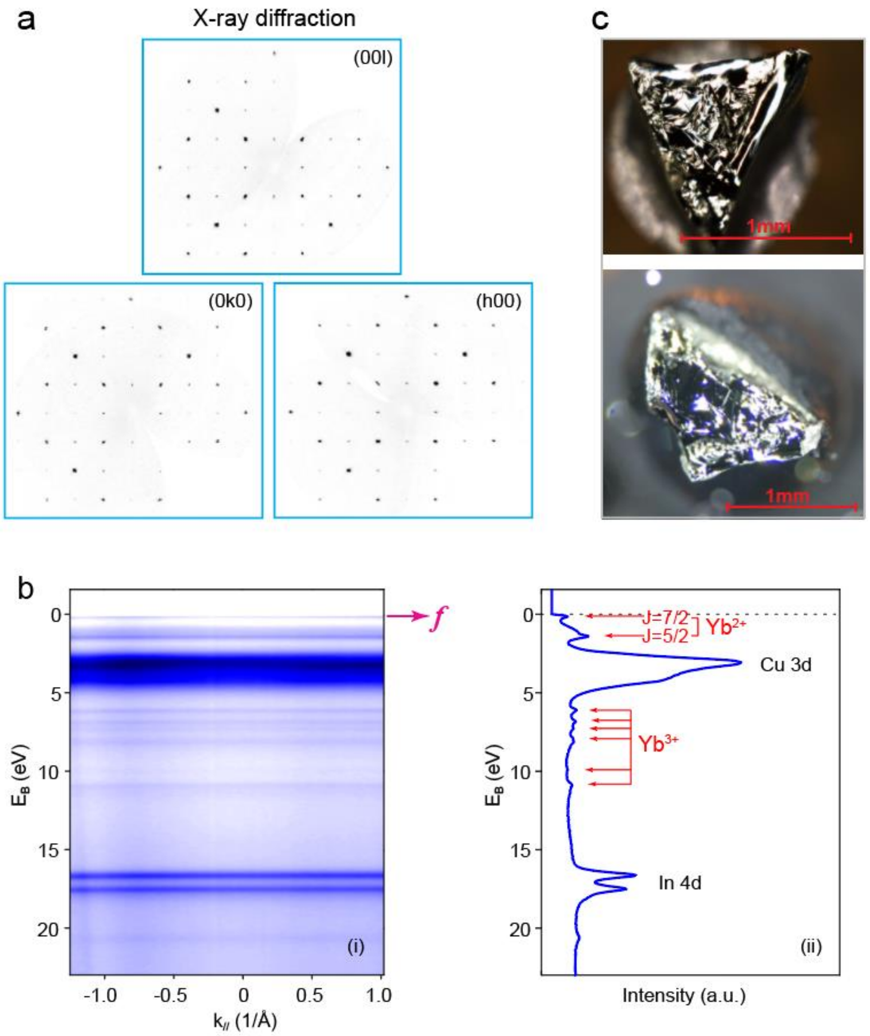

Supplementary Figure 1. Basic characterization of high-quality $YbInCu_4$ crystals. a. X-ray diffraction patterns along (00l), (0k0) and (h00) crystalline directions. $YbInCu_4$ crystallizes in a FCC structure with a lattice constant of a = b = c = 7.17 Å (measured at 150 K). b. Photoemission core-level measurements on $YbInCu_4$ crystals using 100-eV photons. (i) and (ii) are the photoemission image and integrated EDC, respectively. **c**. Optical images of cleaved surfaces of $YbInCu_4$ crystals after ARPES measurements.

In the main text, we have shown Laue diffraction (Fig. 2a) and transport (Fig. 2b) measurements on $YbInCu_4$ crystals, both reflecting the crystals are of high quality, given sharp diffraction spots in the former and narrow transition width in the latter. Here we present X-ray diffraction measurements showing sharp diffraction spots (Supplementary Figure 1a), and ARPES core-level measurements showing sharp peaks of Yb *4f*, Cu *3d*, and In *4d* (Supplementary Figure 1b). These again confirm the high quality of our crystals.

In Supplementary Figure 1b, besides core-level peaks, we have also marked LHB belonging to Yb^{3+} and *f* bands belonging to Yb^{2+} (UHB-*f'* not marked). The *f* band is as flat (non-dispersive) as those core-levels (e.g., In *4d*) in high-binding energy regions.

As $YbInCu_4$ crystallizes in a FCC structure with a rather big unit cell, there exists no natural cleaving planes. We usually failed to get big flat cleaved surfaces but small and fractured surfaces on (111) surface (in more than forty times of experiments), which thus highly necessitates focused-beam ARPES to acquire reliable data.

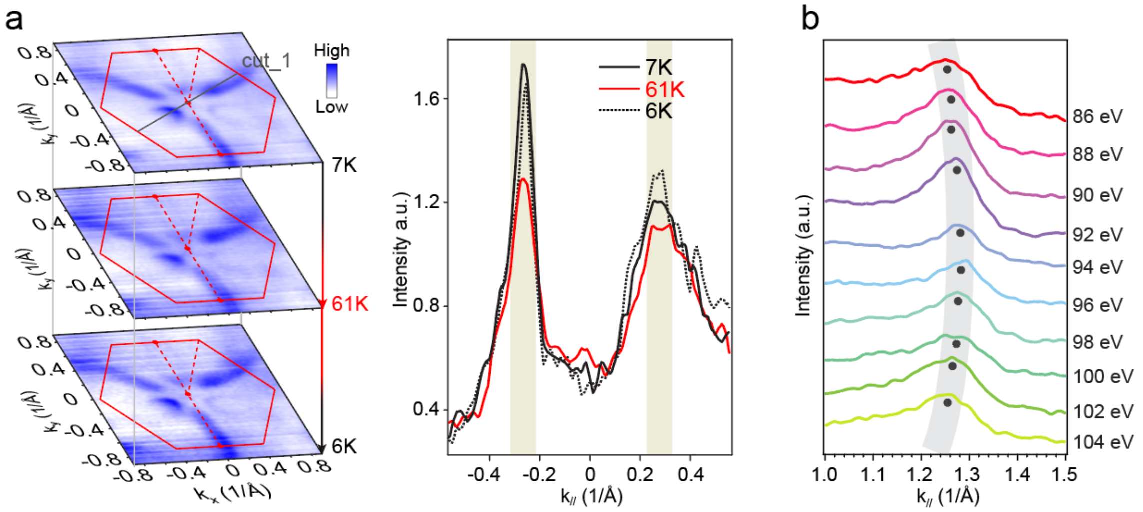

Supplementary Figure 2. Dispersive α bands: Fermi surfaces and bulk state nature. a. Fermi surfaces vertically stacking (left panel), and MDCs at E_F along cut_1 (right panel) measured at 7 K, 61 K, and 6 K, respectively. Stripes are not real band structures but fake features. b. Photon-energy dependence of dispersive α bands. Black circles mark Fermi crossings of the α-band in the second BZ.

In the main text (Figs. 2c and 2d), we have shown that the Fermi surface built-up by dispersive α bands show negligible temperature dependence (across T_V). If cutting the Fermi surface along cut_1 (marked in the left panel of Supplementary Figure 2a), one can further see Fermi crossings vary little with temperature (except being slightly broad at high temperatures) contrasting the dramatic change of Kondo temperature T_K (400 K to 20 K).

We also checked the bulk state nature of α bands by performing photon-energy dependent measurements (Supplementary Figure 2b). 104-86 eV photons were used. Fermi crossings of the α-band (in the second BZ) changes apparently with photon energies (reflecting the k_z dispersion of the α-band), thus confirming its bulk state nature.

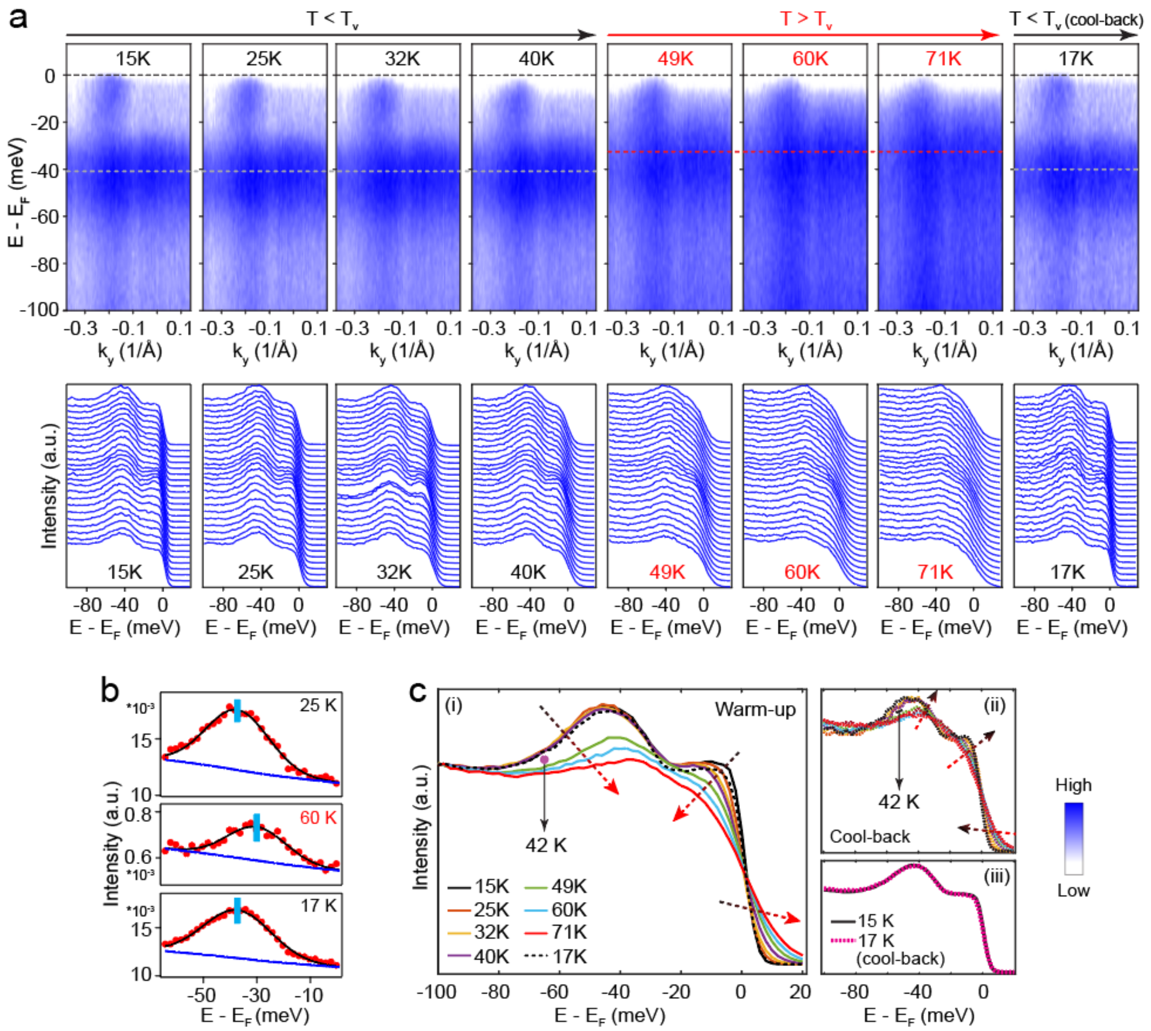

Supplementary Figure 3. Detailed temperature-dependence of near-E_F band structure measured with 6.994-eV laser-ARPES. a. Temperature evolution of the $\bar{\Gamma} - \bar{M}$ dispersion (15 K→71 K→17 K) (upper panel) and their EDC plots (lower panel). Overlaid dotted lines roughly mark binding energy positions of the *f* band. One can apparently see that the *f* band below T_V do not show evident differences until crossing T_V, when *f* band suddenly shifts up. The *f* band shifts down again when cooling through T_V. b. EDC fitting results (25 K, 60 K, and 17 K) show shift-up of *f*-band across T_V. The EDC is extracted around $k_y = 0$ and fitted with a Gaussian. c. Temperature-dependence of integrated EDCs including both α and *f* bands. Warm-up results (i) are well reproduced by a follow-up cool-back (ii). In (i) and (ii), EDCs of both warm-up and cool-back can be easily classified into two groups, well separated by T_V, indicative of the valence transition. In (iii), integrated EDC at 15 K perfectly matches that of 17 K (cool-back) (area-normalization is used), which again verifies our temperature-dependence results are reliable.

In Fig. 3 of the main text, we have shown that *f* band suddenly jumps towards E_F when heating the sample across T_V. Here we show more details of these data in Fig. 3. In Supplementary Figure 3a, we display temperature-dependent dispersion cuts in a flat way (upper panel) with corresponding EDC plots (lower panels). Related to Fig. 3b of the main text, we show three representative EDC fitting results (at 25 K, 60 K, and 17 K), clearly showing the *f* band moves close to E_F above T_V (Supplementary Figure 3b).

In Supplementary Figure 3c, we present temperature-dependence of integrated EDCs including both α and *f* band near E_F. Apparently, these EDCs can be classified into two groups, well separated by T_V = 42 K. Note that these warm-up results are reliable as confirmed by a follow-up cool-down experiments (Supplementary Figure 3c(ii)). Besides, integrated EDC at 15 K perfectly matches that of 17 K (cool-back) (area-normalization is used) (Supplementary Figure 3c(iii)).

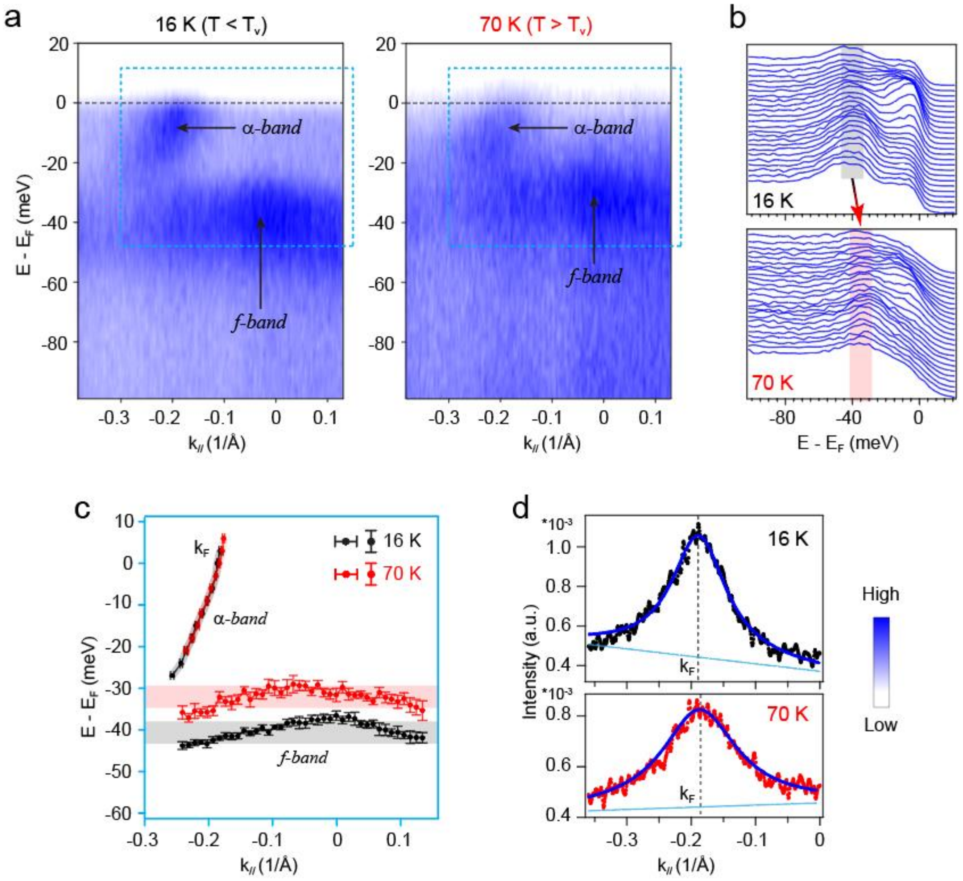

Supplementary Figure 4. Laser-ARPES measurements on another $YbInCu_4$ crystal. a. Band dispersions along $\bar{\Gamma} - \bar{M}$ at 16 K (left; < T_v) and 70 K (right; > T_v), both clearly show α and *f* bands. b. EDC plots of band dispersions in a. Arrow marks the jump of the *f* band. c.

Extracted α and f band dispersions at 16 K (in black) and 70 K (in red) by EDC/MDC fitting with a Gauss/Lorentz function plus a linear background. While α-band exhibits negligible changes, the f band jumps towards high-binding-energy regions. Seemingly slight dispersions of f-band is due to fitting process. d. MDC at E_F (at 16 K and 70 K) fitted with a Lorentz function plus a linear background. Fermi crossing point (namely Fermi momentum k_F) almost remains unchanged.

In Supplementary Figure 4, we present laser-ARPES measurements on another $YbInCu_4$ crystal. From raw images, corresponding EDC plots and extracted dispersions by EDC/MDC fittings, one can clearly the f band jumps towards high-binding-energy regions cooling through T_V. By contrast, the α-band exhibits negligible changes, in line with observations that Fermi surfaces almost remains unchanged in Fig. 2 of the main text and Supplementary Figure 2.

Hybridization seems to occur between the α and f-bands (Supplementary Figure 4). However, we could not track the α band in a convincing way when it approaches the f band, neither did we know whether the gap truly forms or the α band loses some spectral weight. The closing of "hybridization gap" at high temperatures (e.g., 70 K) can be alternately explained by the abrupt jump-up of the f-band. Thus, we decided not to describe the feature as a hybridization gap.

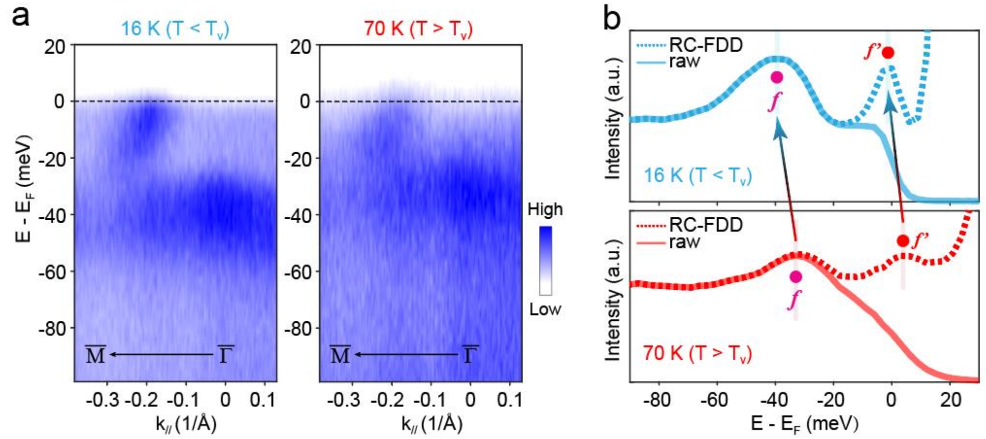

Supplementary Figure 5. Observation of the f' band (UHB) and Mott transition across T_V. a. Band dispersions along $\bar{\Gamma} - \bar{M}$ at 16 K (left; < T_v) and 70 K (right; > T_v). b. Integrated EDCs (integration window ~ [-0.1, 0.1Å^{-1}]) and their RC-FDD results, unambiguously reveal the f' band does exist and shift down to cross E_F when cooling the system through T_V (as marked by arrows).

Based on the data shown in Supplementary Figure 4, by means of RC-FDD, the *f'* band (UHB) is evidently revealed. (Even in the raw image below T_V (Supplementary Figure 5a: left panel), one can see residue spectral weight at E_F). When cooling through T_V, both *f* and *f'* bands move towards high-binding-energy regions; moreover, the *f'* band moves to E_F, thus realizing an orbital-selective Mott transition responsible for abrupt changes of physical properties of $YbInCu_4$.

Experimentally, it is feasible to capture detailed temperature evolution of the *f*-band (located ~ 40 meV below E_F) via ARPES; but is rather hard to do the same thing towards the *f'* band (located slightly above E_F). We argue that *f* and *f'* should act together in response to the VT-related charge transfer. Thus, the movement of the *f'* band should be intimately related to the VT in $YbInCu_4$ as well.

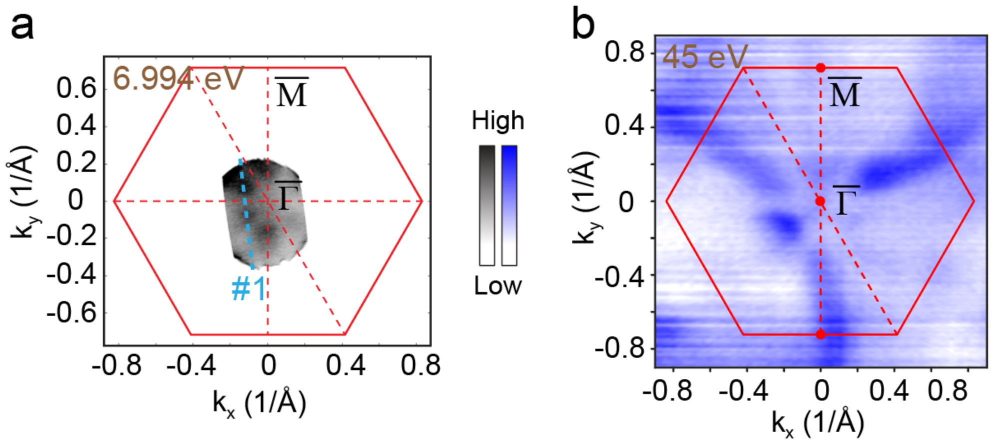

Supplementary Figure 6. Fermi surface maps acquired with 6.994-eV laser (a) and 45-eV (synchrotron light source) (b). Band dispersions in Fig. 4a(i) of the main text are measured along #1 (marked in a).

In Supplementary Figure 6, we present Fermi surface map acquired with Laser-ARPES that shows six ellipse patches stretching towards the Γ point. This is similar to that obtained by synchrotron–based ARPES (45 eV).

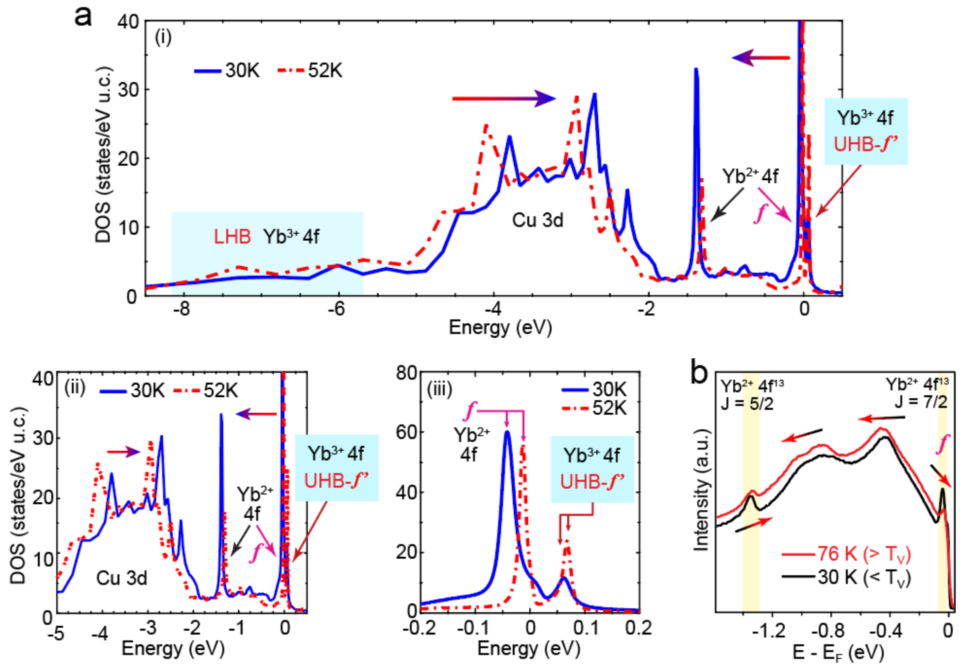

Supplementary Figure 7. Valence transition related charge transfer reflected by shifts of various core-level peaks and spectral weight transfer. a. Calculated DOSs at 30 K (below T_V, solid in blue) and 52 K (above T_V, dotted in red). Peaks of Yb^{2+}/Yb^{3+} and LHB/UHB are marked. b. Integrated EDCs measured at 30 K (in black) and 76 K (in red) with 21.2-eV photons.

In the main text, we have shown valence-change-related charge transfer serves as self-doping to drive the orbital-selective Mott transition. Detailed calculation results of density of states above/below T_V is presented in Supplementary Figure 7a. When cooling through T_V, charge transfer occurs in a quite wide binding-energy region, manifested by shifts of core-level peaks and spectral weight transferring. This is confirmed by our ARPES measurements (Supplementary Figure 7b), hard X-ray photoemission experiments [1, 2] and RIXS [3] results reported previously (e.g., Cu $3p_{3/2}$ and In $3d_{5/2}$ are shifted by ~40 and ~30 meV towards higher-binding energy when heating across T_V [2], respectively, while Yb^{3+} *4f* (~6.0 eV) moves oppositely by 65 meV [1]). Such drastic change of the spectra naturally reflects the valence change across T_V, which would in turn trigger the Mott transition in the Yb^{3+} band.

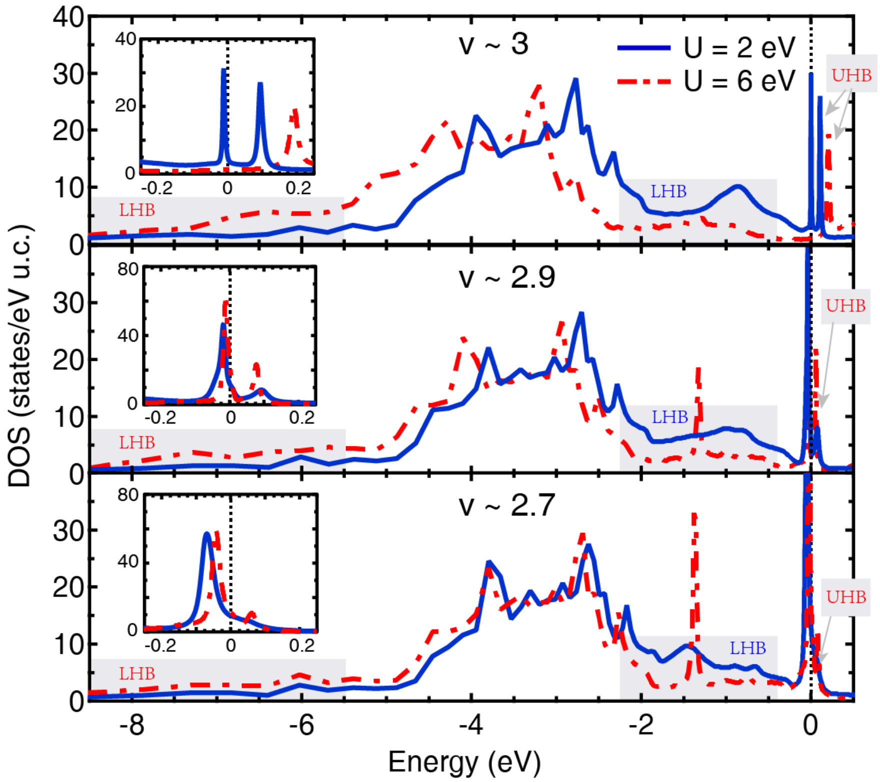

Supplementary Figure 8. Comparison of the total density of states for U = 2, 6 eV and $v \approx$ +3, 2.9, 2.7 at 30 K. The LUB and UHB are indicated for clarity. The insets show the enlarged plots near E_F.

To identify the origins of different DOS peaks, we compare their respective variation with the Coulomb interaction U for different average Yb valence $v \approx$+3, 2.9, and 2.7 (Supplementary Figures 8 and 9). Deviation from integer valence may be viewed as a mixture of Yb^{2+} and Yb^{3+} states (the true valence of Yb ions is close but not identical to +2 and +3 according to the simple ionic picture, and there is no spatial separation of Yb^{2+} and Yb^{3+} sites). The Coulomb interaction plays the role of tuning the Kondo coupling [4]. The temperature is fixed to avoid confusion due to thermal broadening. At U = 6 eV, the peak right below the Fermi energy disappears for v = +3 but remains sharp for other two valences, suggesting that the former comes from the Kondo quasi-particles, while the latter two are the fully occupied *4f* bands of Yb^{2+} ions. The peak above the Fermi energy corresponds to the hole band (UHB) of Yb^{3+} and its position changes only slightly with U, while the broad LHB is located at roughly –U and moves accordingly. The Yb^{2+} bands are well described by pure DFT calculations with proper renormalization. As v decreases, Yb^{2+} bands grow

stronger and the broad UHB shifts downward to touch the Fermi energy, as implied from the insets. These features are in qualitative (or quantitative) agreement with experiments, thus providing a most probable interpretation of the key ARPES features supporting the proposed mechanism, although the Yb^{2+} bands in calculations are not as flat as that in experiment and exact positions of the calculated UHB and Yb^{2+} bands are also slightly higher in energy. It is noteworthy that we did not mean to claim that the Kondo physics is completely absent. Incoherent Kondo scattering is indeed seen above T_V in the insulating-like resistivity. It is simply that the true Kondo temperature is too small (~20 K) and the Kondo hybridization is too weak to have an evident effect on the band structures (or a Kondo resonance in the DOS).

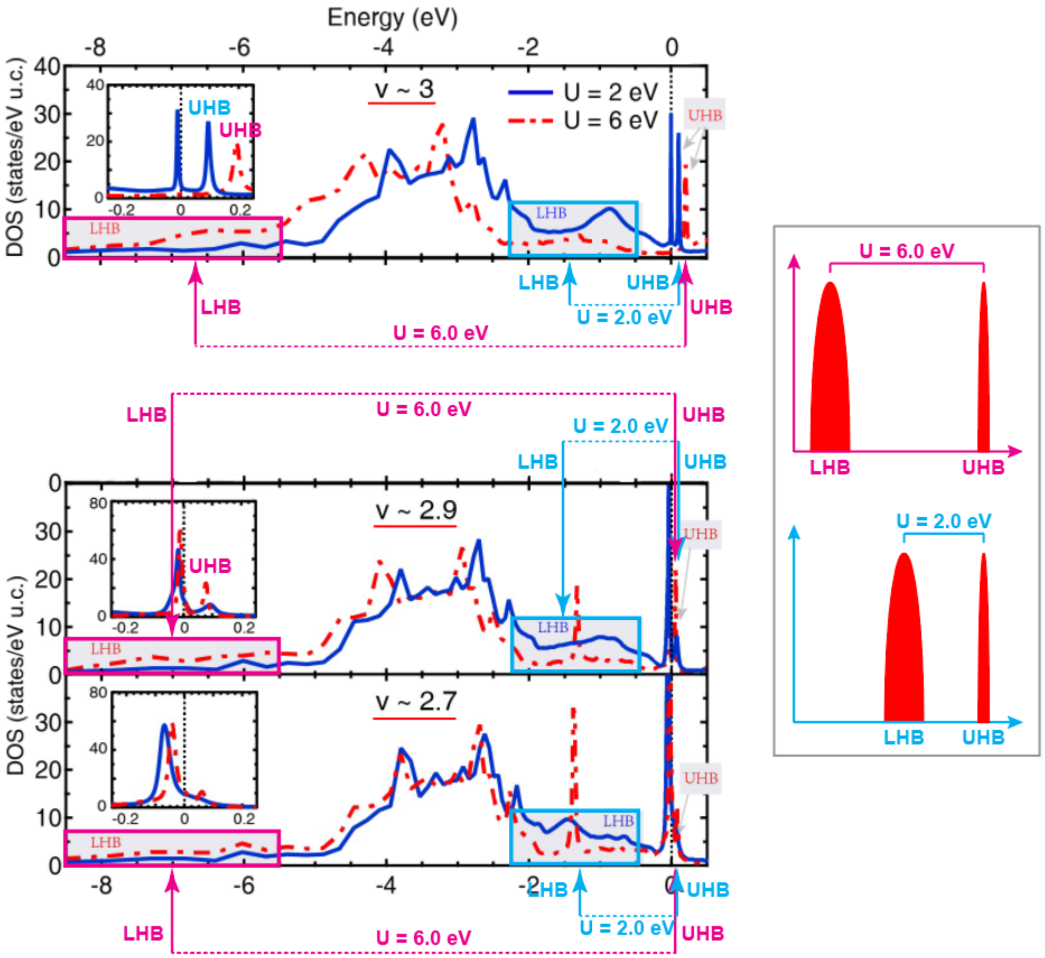

Supplementary Figure 9. Similar to Supplementary Figure 8 but with more markers and a schematic of LHB and UHB (right).

Supplementary Figure 10 presents more detailed analysis of the orbital-resolved spectra. Apparently, the two peaks near the Fermi energy are from Yb *4f* orbitals (Yb^{3+} UHB and Yb^{2+}) with $J = 7/2$ and the conduction spectra are mainly from Cu *d* orbitals. Although no Kondo resonance is revealed, there does exist usual hybridization between Yb 4*f* and Cu *d* bands, as the Cu *d* partial DOS has similar peak structure at the Yb *4f* peaks (Yb^{3+} UHB and Yb^{2+}). Actually, solely from ARPES data, we can also rule out the Kondo resonance as the origin of *f* and *f'* bands: above T_V (42 K), $YbInCu_4$ is shown to possess a T_K of ~ 20 K; if *f* or *f'* bands are indeed of Kondo resonance origin, they should disappear at a temperature well above such T_K, under the context of the Kondo physics; however, *f* and *f'* bands can persist up to 70 K (> 20 K, even to 100 K in our measurements).

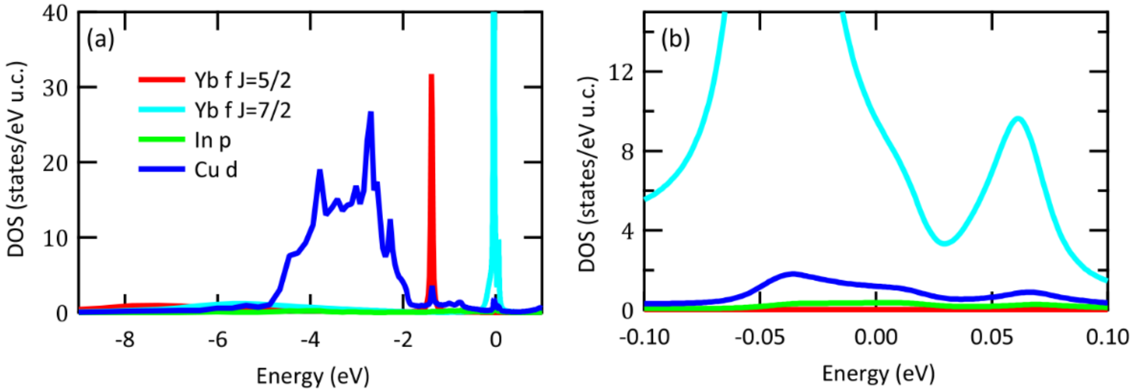

Supplementary Figure 10. Orbital-resolved partial density of states for U = 6.0 eV and T = 30 K within different energy ranges in (a) and (b).

[1] S. Suga, A. Sekiyama, S. Imada, J. Yamaguchi, A. Shigemoto, A. Irizawa, K. Yoshimura, M. Yabashi, K. Tamasaku, A. Higashiya, and T. Ishikawa. Journal of the Physical Society of Japan **78**, 074704 (2009).

[2] Y. Utsumi, H. Sato, H. Kurihara, H. Maso, K. Hiraoka, K. Kojima, K. Tobimatsu, T. Ohkochi, S. Fujimori, Y. Takeda, Y. Saitoh, K. Mimura, S. Ueda, Y. Yamashita, H. Yoshikawa, K. Kobayashi, T. Oguchi, K. Shimada, H. Namatame, and M. Taniguchi, Phys. Rev. B **84**, 115143 (2011).

[3] I. Jarrige, A. Kotani, H. Yamaoka, N. Tsujii, K. Ishii, M. Upton, D. Casa, J. Kim, T. Gog, and J. N. Hancock, Phys. Rev. Lett. **114**, 126401 (2015).

[4] A. C. Hewson. The Kondo Problem to Heavy Fermions. Cambridge University Press, Cambridge, England, 1997.
